# Integrated DNA Methylation and Gene Expression Analysis Identified S100A8 and S100A9 in the Pathogenesis of Obesity

**DOI:** 10.3389/fcvm.2021.631650

**Published:** 2021-05-13

**Authors:** Ningyuan Chen, Liu Miao, Wei Lin, Donghua Zou, Ling Huang, Jia Huang, Wanxin Shi, Lilin Li, Yuxing Luo, Hao Liang, Shangling Pan, Junhua Peng

**Affiliations:** ^1^Department of Pathophysiology, School of Preclinical Medicine, Guangxi Medical University, Nanning, China; ^2^Department of Cardiology, Liuzhou People's Hospital, Guangxi Medical University, Liuzhou, China; ^3^Department of Neurological Rehabilitation, Guangxi Jiangbin Hospital, Nanning, China; ^4^Department of Neurology, The Fifth Affiliated Hospital of Guangxi Medical University, Nanning, China; ^5^The First Clinical Medical School, Guangxi Medical University, Nanning, China

**Keywords:** obesity, DNA methylation, mRNA expression, function enrichment, correlation analyses

## Abstract

**Background:** To explore the association of DNA methylation and gene expression in the pathology of obesity.

**Methods:** (1) Genomic DNA methylation and mRNA expression profile of visceral adipose tissue (VAT) were performed in a comprehensive database of gene expression in obese and normal subjects. (2) Functional enrichment analysis and construction of differential methylation gene regulatory networks were performed. (3) Validation of the two different methylation sites and corresponding gene expression was done in a separate microarray dataset. (4) Correlation analysis was performed on DNA methylation and mRNA expression data.

**Results:** A total of 77 differentially expressed mRNAs matched with differentially methylated genes. Analysis revealed two different methylation sites corresponding to two unique genes—s100a8-cg09174555 and s100a9-cg03165378. Through the verification test of two interesting different expression positions [differentially methylated positions (DMPs)] and their corresponding gene expression, we found that methylation in these genes was negatively correlated to gene expression in the obesity group. Higher S100A8 and S100A9 expressions in obese subjects were validated in a separate microarray dataset.

**Conclusion:** This study confirmed the relationship between DNA methylation and gene expression and emphasized the important role of S100A8 and S100A9 in the pathogenesis of obesity.

## Introduction

With continuous improvement of living conditions, many countries have to pay more attention to the prevalence of obesity because obesity has reached the proportion of epidemic that is still rising (1). According to the World Health Organization's report in 2015, about one third or more adults are overweight, 43% of whom are male and 45% are female (2). Obesity is implicated in many diseases, such as metabolic syndrome (MetS), type 2 diabetes mellitus (T2DM), hypertension, arteriosclerosis, cardiovascular disease (CVD), and so on (3), and thus incurs heavy economic burdens on countries around the world (4). Obesity results from interactions between genetic and environmental factors, but for individuals, epigenetic factors can increase susceptibility to obesity (5).

In recent years, with the deepening of research, epigenetics has emerged as a bridge between genes and environmental factors. It can change gene expression and induce long-term changes in phenotype and disease susceptibility (6). Extensive epigenome-wide association studies (EWAS) on DNA methylation conducted in many populations have revealed the relationship between DNA methylation and obesity (7). Changes in gene methylation changes can alter the transcription of genes resulting in abnormal gene expression and eventually obesity (8).

Gene Expression Omnibus (GEO) database hosted the sequencing data of thousands of researchers, and its most important feature was that it provided open access to the data we needed to conduct our research. In the present study, we explored innovative methylation sites of obesity-related DNA by conducting an integration study using two microarray datasets and established the relationship between the expression of obesity-related genes and methylation Differential Expression of Methylated Genes (DEMGs). We then validated the DEMGs in a separate microarray dataset to explore the potential relationship between DNA methylation and mRNA expression on the regulation of obesity.

## Methods

### Gene Expression Profile and Probe Labeling

Three microarray datasets (GSE88837, GSE88940, and GSE109597) were downloaded from the gene expression database (https://www.ncbi.nlm.nih.gov/geo/) for analysis. GSE88837 was extracted from the U133 + 2.0 sequence of gpl570 Affymetrix human genome for gene expression. The purpose of this study was to use global gene expression to identify obesity-induced changes in gene expression profiles of lean and obese adolescent females. In our study, subjects with body mass index (BMI) ≥30 were defined as obese (1). A total of 30 subjects (including 15 obese and 15 healthy controls) were analyzed. We used the Affy package in R (9) to convert the cel files into an expression value matrix and the Robust Multichip Average (RMA) method to normalize the matrix. The Bioconductor package in R software was used to convert probe data into genes (10). If a gene corresponded to several probes, we chose the average expression value for further analysis. GSE88940 extracted from gpl13534, a human methylation 450 gene chip, was used for DNA methylation analysis, which consisted of 10 objects and 10 lean controls. Genomic DNA was extracted from visceral adipose tissue (VAT) of lean and obese adolescent females. Illumina Infinium Human Methylation 450 k BeadChips were utilized for global methylation profiles in VAT. All data processing was done in GEO2R (https://www.ncbi.nlm.nih.gov/geo/geo2r/). In these two datasets (GSE88837 and GSE88940), there are 20 samples of the same person. We only analyze these 20 samples. GSE109597 was used as the validation dataset and aimed to predict obesity risk with genetic data, specifically, obesity-associated gene expression profiles. Genetic risk score was computed. The genetic risk score was significantly correlated with BMI when an optimization algorithm was used. Linear regression and built support vector machine models predicted obesity risk using gene expression profiles and the genetic risk score with a new mathematical method. The analysis method was the same as used for GSE 88837.

### Differential Expression and Analysis of Methylated Genes (DEMGs)

We compared obese with control subjects to explore the differentially expressed genes (DEGs) of the marginal envelope in R (11). The threshold value was set as | log2 fold change |≥ 2, *P* < 0.05. GEO2R was used to determine the methylation sites [differentially methylated positions (DMPs)] by comparing the differences between normal and obese subjects. DMPs located in gene regions were assigned to corresponding genes, which were defined as differentially methylated genes (DMGs). The threshold value was set as |log_2_ fold-change| (Δβ) > 0.05, *P* < 0.05. Then, we matched the DEGs with DMGs, and only the matched genes (DEMGs) were selected for further analysis.

### Functional Enrichment Analysis

All functional enrichment analysis on DEGs was performed on clusterProfiler and Dose package in R (12). The complete functional enrichment analysis includes Gene Ontology (GO), Kyoto Encyclopedia of Genes and Genomes (KEGG) approach, and Disease Ontology (DO). The threshold value of analysis was set as adjust-*P* < 0.05 and error detection rate [false discovery rate (FDR)] <0.05.

### Protein–Protein Interaction Network and Module Analysis

We used the string database (version 11.0) (13) to explore protein prediction and experimental interactions. There are many methods of database prediction, including co-expression experiment, text mining, co-occurrence, gene fusion, database, and neighborhood. Also, we used the combination fraction to reveal the protein pair interactions in the database. Then, we localized DEMGs to PPIs to identify the key genes in the network with the cutoff value set to a comprehensive score >0.9 (14). As a valuable method, a degree is used to study the role of protein nodes in the network. Using the molecular complex detection (MCODE) on Cytoscape (version 3.71), the most significant clustering module and the main clustering module were explored (15, 16). For further analysis, we set ease ≤ 0.05 and set ≥2 as the cutoff value and MCODE score >8 as the threshold value.

### Validation of DEMGs

We used prism 8.0 GraphPad (17) for scatterplots of methylation and gene expression to detect the relationship between methylation and gene expression. Then, we calculated the correlation equation to judge whether the equation has statistical significance. The DEMGs were validated in GSE109597, which contained 84 unrelated samples. After grouping according to BMI (>30 and <30), the expression of DMEGs in the two groups was compared with ggplot2 in R.

## Results

### Data Preprocessing

**Figure 1:**
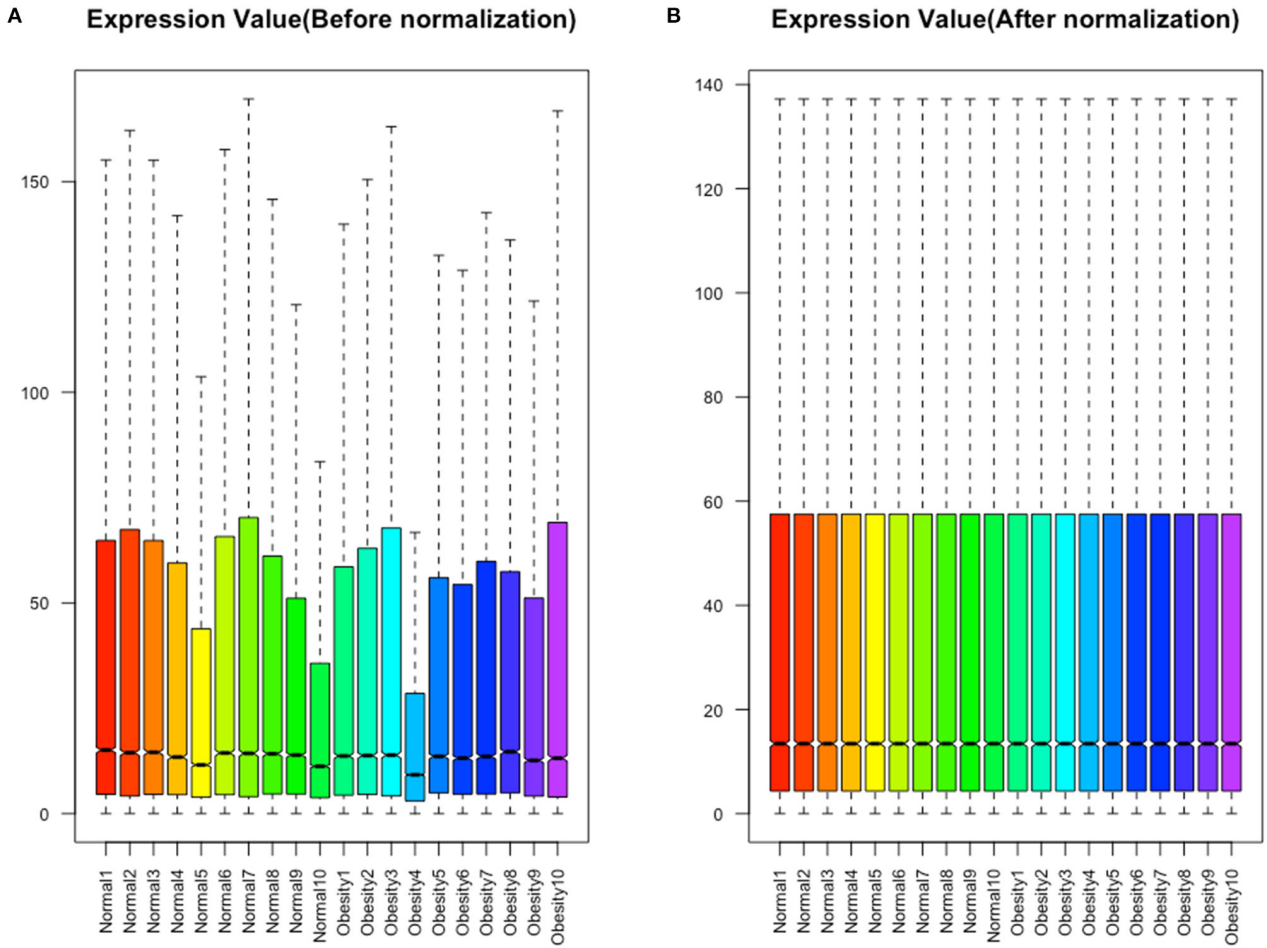
Normalization of all samples. (**A**) Before normalization. (**B**) After normalization.

**Figure 2:**
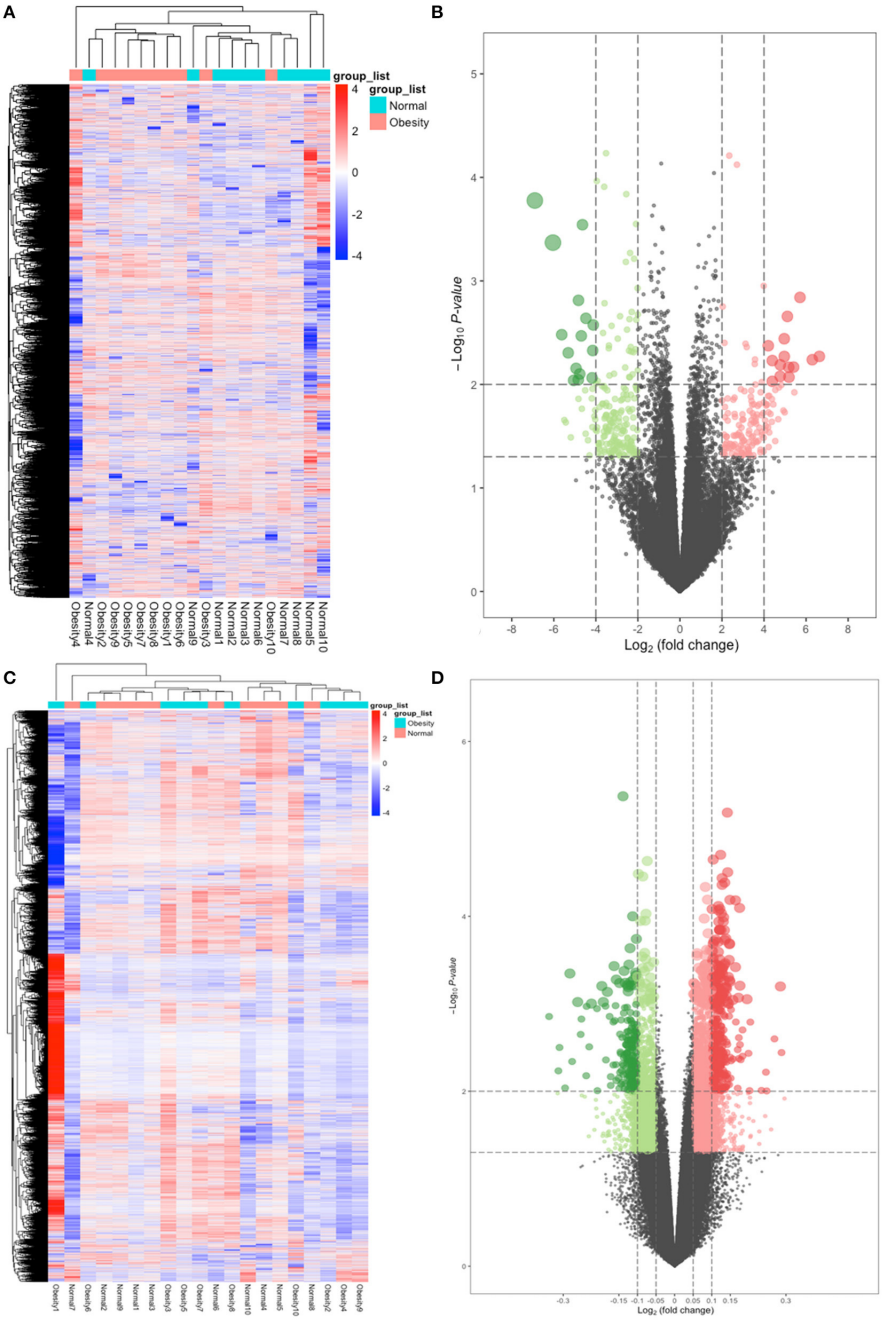
Heatmap and volcano plots of differentially expressed genes (DEGs) and differentially methylated positions (DMPs). **(A)** Heatmap for DEGs. Obesity groups are in the red cluster, and normal samples are in the green cluster. **(B)** Volcano plot for DEGs. The two vertical lines are the 2-fold change boundaries, and the horizontal line is the statistical significance boundary (*P* < 0.05). Red dots show upregulated genes, and green dots are downregulated genes. **(C)** Heatmap for DMPs. Obesity groups are in the green cluster, and normals are in the blue cluster. **(D)** Volcano plot for DMPs. The vertical lines are the 0.05-fold change boundaries, and the horizontal line is the statistical significance boundary (*P* < 0.05). Upregulated DMPs are marked with red dots, and downregulated DMPs are marked with green dots.

A quality control processing of GSE88837 showed that when all the means in the strip chart lie on the same horizontal line, all samples were normalized (Figure 1). We obtained 54,560 expression probes from each gene map and expression. Probes with too low or too high probe expression were defined as outliers and eliminated from further analysis. An average expression value was used to screen the DEGs to prevent too many probes corresponding to one gene. The limma software package was used to calculate the DEGs, which yielded 1,814 DEGs with *P* values <0.05. We use the obese population as a reference, among these, 150 with a log2 (fold change) > 2 were defined as upregulated and 199 with a log2 (fold change) <2 as downregulated. The heat map and volcano map of DEGs are shown in Figures 2A,B.

Out of the 485,579 DNA methylation sites in GSE88940 VAT that were screened for quality control, 454,325 methylation sites were selected for analysis. Here, 10,016 DMPs (|Δβ| > 0.05, *P* < 0.05) were identified, of which 666 were hypermethylated and 3,349 were hypomethylated. After annotation, 10,016 DMPs are located in 4,024 unique genes, which were identified as DMGs. The thermal and volcanic maps of the DMGs are shown in Figures 2C,D.

**Figure 3:**
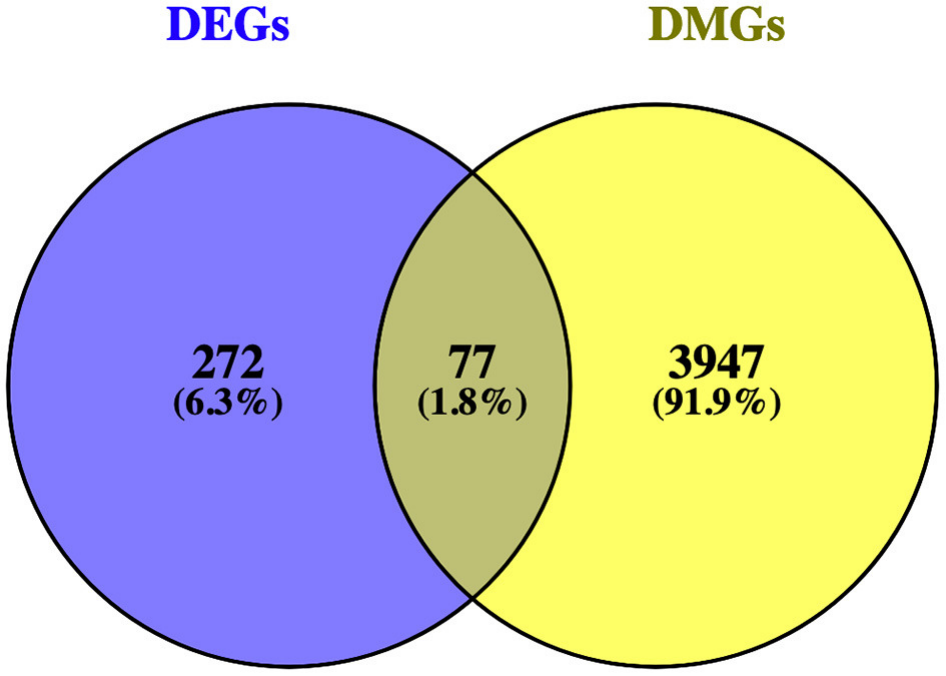
Venn diagram showing the intersection of differentially expressed genes (DEGs) and differentially methylated genes (DMGs).

**Figure 4:**
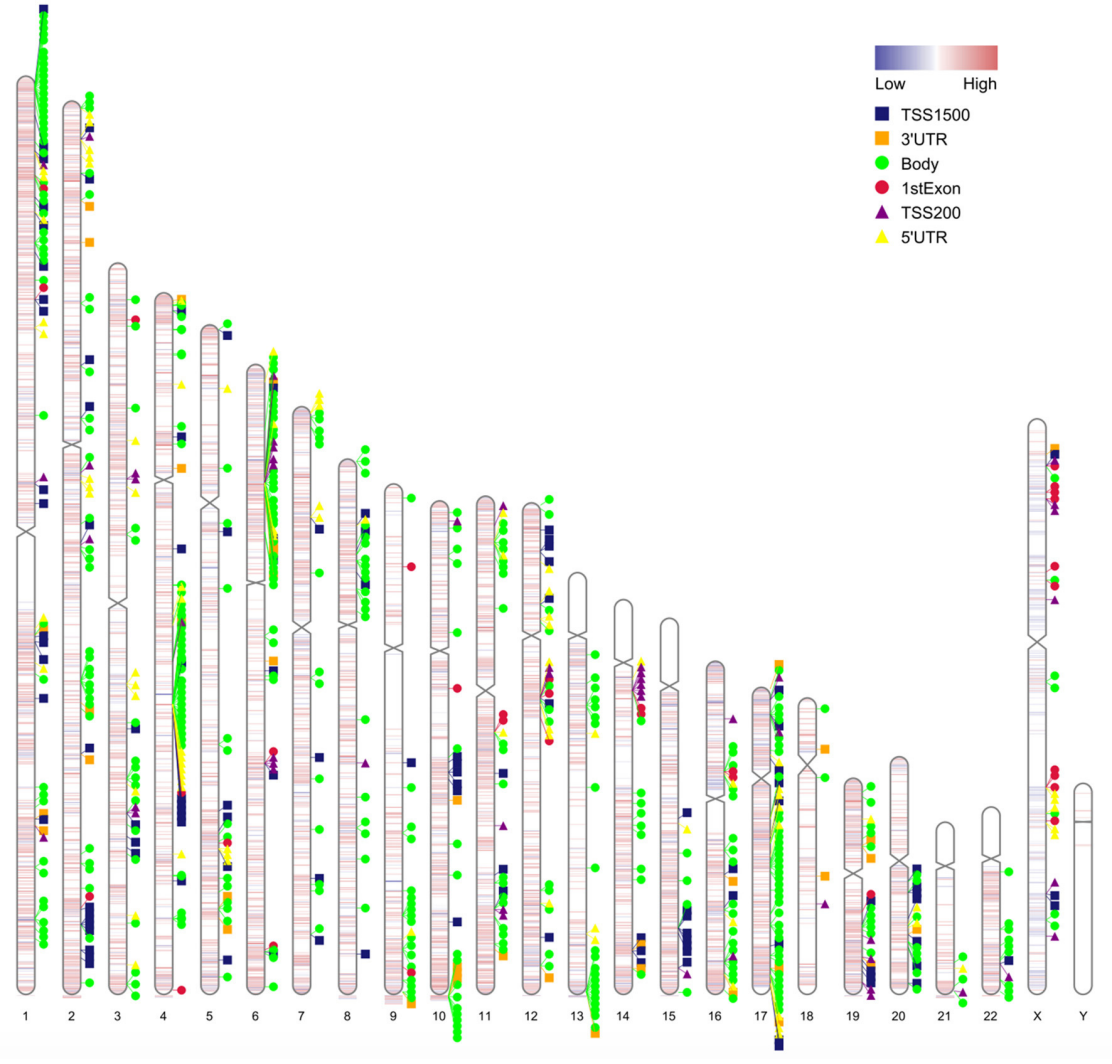
Chromosome distribution of differentially methylated intergenic CpGs. Plot showing the distribution of differential intergenic CpG sites on 22 autosomes and the X and the Y chromosomes. Red is the hypermethylated region and blue is the hypomethylated region with logFC values of the M value between obesity patients and healthy controls.

Matching DMGs with DEGs yielded 77 genes for the next analysis (Figure 3). The details of 77 genes are shown in Table 1 and Figure 4.

**Table 1:** The matched pairs of DEG and DMP.

**DMPs**								**DEGs**	
**SYMBOL**	**CpG site**	**START**	**END**	**CHR**	**Position**	**Δβ**	***P* values**	**FC**	***P* values**
ADH4	cg12011299	100065546	100065669		TSS200	7.95E-02	1.13E-02	−4.15	4.70E-03
ASPHD1	cg05192831	29913007	29913130	16	1stExon	5.42E-02	1.13E-02	−5.50	2.21E-02
ASPHD1	cg02488299	29913223	29913346	16	1stExon	6.23E-02	1.13E-02	−5.50	2.21E-02
BNC1	cg27169020	83954229	83954352	15	TSS1500	7.85E-02	1.13E-02	−2.54	3.92E-02
BNC1	cg23741520	83954231	83954354	15	TSS1500	7.86E-02	1.13E-02	−2.54	3.92E-02
BNC1	cg12250049	83951663	83951786	15	Body	8.56E-02	1.13E-02	−2.54	3.92E-02
BNC1	cg10275315	83954246	83954369	15	TSS1500	9.20E-02	1.13E-02	−2.54	3.92E-02
BNC1	cg00768409	83954392	83954515	15	TSS1500	9.23E-02	1.13E-02	−2.54	3.92E-02
BNC1	cg16049391	83954395	83954518	15	TSS1500	1.06E-01	1.13E-02	−2.54	3.92E-02
C1orf87	cg17238766	60539675	60539798	1	TSS1500	8.87E-02	1.13E-02	3.84	1.19E-02
C1orf87	cg09851465	60539671	60539794	1	TSS1500	1.07E-01	1.13E-02	3.84	1.19E-02
C3orf22	cg07743179	126278943	126279066	3	TSS1500	−5.14E-02	1.13E-02	2.63	4.95E-02
CFB	cg12124018	31916683	31916806	6	Body	−6.73E-02	1.13E-02	−3.60	1.23E-04
CFB	cg21606287	31916705	31916828	6	Body	−6.17E-02	1.13E-02	−3.60	1.23E-04
CGN	cg07596668	151509825	151509948	1	3'UTR	8.20E-02	1.13E-02	−4.83	1.38E-02
CGN	cg25198049	151509723	151509846	1	Body	8.29E-02	1.13E-02	−4.83	1.38E-02
CHI3L1	cg14085262	203155938	203156061	1	TSS200	5.79E-02	1.13E-02	−3.09	1.38E-02
CRB2	cg15922174	126135998	126136121	9	Body	−8.61E-02	1.13E-02	−4.69	3.40E-03
CRB2	cg02625222	126135169	126135292	9	Body	5.30E-02	1.13E-02	−4.69	3.40E-03
CRB2	cg11431402	126135408	126135531	9	Body	5.76E-02	1.13E-02	−4.69	3.40E-03
CRB2	cg13884995	126126377	126126500	9	Body	7.19E-02	1.13E-02	−4.69	3.40E-03
DMRTA1	cg14338345	22447679	22447802	9	1stExon	5.36E-02	1.13E-02	4.92	1.79E-02
DUSP1	cg08452061	172199642	172199765	5	TSS1500	5.88E-02	1.13E-02	5.41	6.80E-03
EPHA1	cg21294616	143093848	143093971	7	Body	−6.88E-02	1.13E-02	3.61	3.53E-02
EPHA1	cg26960083	143106298	143106421	7	TSS1500	6.90E-02	1.13E-02	3.61	3.53E-02
ESPN	cg13284574	6519923	6520046	1	Body	7.33E-02	1.13E-02	4.19	2.59E-02
FAM84A	cg12050497	14773274	14773397	2	5'UTR	9.44E-02	1.13E-02	−4.84	9.04E-03
FGF9	cg03688324	22251017	22251140	13	Body	6.28E-02	1.13E-02	−2.68	1.25E-02
FNDC1	cg06764804	159654028	159654151	6	Body	5.46E-02	1.13E-02	−3.50	1.97E-02
GPR143	cg19318920	9693690	9693813	X	3'UTR	5.73E-02	1.13E-02	−2.33	3.60E-02
GPRIN3	cg02734358	90227074	90227197	4	5'UTR	1.06E-01	1.13E-02	−4.03	2.18E-02
GSC	cg01695643	95237330	95237453	14	TSS1500	8.16E-02	1.13E-02	2.62	3.78E-02
GSC	cg15440688	95237637	95237760	14	TSS1500	9.33E-02	1.13E-02	2.62	3.78E-02
ITGA2B	cg14686645	42452426	42452549	17	Body	6.75E-02	1.13E-02	2.59	1.34E-02
KCNK3	cg19115882	26919145	26919268	2	Body	5.71E-02	2.04E-03	−2.31	4.83E-03
KCNK3	cg19991086	26953767	26953890	2	3'UTR	6.04E-02	2.04E-03	−2.31	4.83E-03
KCNN3	cg16296829	154832535	154832658	2	TSS200	6.21E-02	2.04E-03	−2.31	4.83E-03
KCNN3	cg18315680	154833117	154833240	2	Body	8.39E-02	2.04E-03	−2.31	4.83E-03
KLF2	cg02668248	16437789	16437912	19	Body	−7.35E-02	2.04E-03	3.95	1.90E-02
KLHL34	cg20312916	21676605	21676728	X	TSS200	−6.58E-02	2.04E-03	3.99	1.49E-02
KLHL34	cg01828474	21676593	21676716	X	TSS200	−5.27E-02	2.04E-03	3.99	1.49E-02
KLHL34	cg25075572	21673930	21674053	X	1stExon	−5.05E-02	2.04E-03	3.99	1.49E-02
KRT7	cg25313172	52627272	52627395	12	1stExon	−6.67E-02	2.04E-03	−3.94	2.60E-02
KRT7	cg14537533	52626904	52627027	12	TSS200	−6.14E-02	2.04E-03	−3.94	2.60E-02
KRT7	cg07967679	52626814	52626937	12	TSS200	−5.58E-02	2.04E-03	−3.94	2.60E-02
KRT7	cg07022048	52638592	52638715	12	Body	6.70E-02	2.04E-03	−3.94	2.60E-02
KRT71	cg23767977	52947465	52947588	15	TSS1500	5.19E-02	2.04E-03	3.47	3.17E-02
KRT8	cg24504361	53297987	53298110	12	Body	6.83E-02	2.04E-03	−2.20	4.88E-03
LAD1	cg11418783	201369650	201369773	1	TSS1500	5.47E-02	2.04E-03	2.97	1.95E-02
MARCO	cg07554474	119698443	119698566	2	TSS1500	6.99E-02	2.04E-03	−2.45	1.73E-02
MC2R	cg25924472	13884152	13884275	18	3'UTR	−1.15E-01	2.04E-03	4.96	3.62E-03
MFAP4	cg15119221	19290755	19290878	17	TSS1500	6.26E-02	2.04E-03	2.22	3.19E-02
MYT1L	cg14128411	1926907	1927030	2	Body	−1.08E-01	2.04E-03	−2.32	4.16E-02
MYT1L	cg00067742	1926888	1927011	2	Body	−7.81E-02	2.04E-03	−2.32	4.16E-02
MYT1L	cg09022325	1796228	1796351	2	Body	5.95E-02	2.04E-03	−2.32	4.16E-02
MYT1L	cg22388316	2000192	2000315	2	5'UTR	8.38E-02	2.04E-03	−2.32	4.16E-02
NGFR	cg04466214	47581280	47581403	17	Body	5.49E-02	2.04E-03	−2.34	4.35E-03
NGFR	cg17369032	47590326	47590449	17	Body	7.93E-02	2.04E-03	−2.34	4.35E-03
PCDHB3	cg01925738	140480770	140480893	5	1stExon	−7.09E-02	2.04E-03	3.59	1.00E-02
PKD2L2	cg10535132	137224284	137224407	5	TSS1500	6.68E-02	2.04E-03	−3.16	2.52E-02
ROR2	cg14244439	94561075	94561198	9	Body	5.75E-02	2.04E-03	−2.71	2.79E-02
ROR2	cg02785332	94647670	94647793	9	Body	6.55E-02	2.04E-03	−2.71	2.79E-02
RXFP1	cg03875996	159442782	159442905	4	TSS1500	7.56E-02	2.04E-03	4.77	6.47E-03
S100A8	cg09174555	153364020	153364143	1	TSS1500	7.03E-02	2.04E-03	−2.25	8.00E-03
S100A9	cg03165378	153329882	153330005	1	TSS1500	7.51E-02	2.04E-03	−2.05	1.13E-02
SCTR	cg26009192	120282200	120282323	2	TSS200	−5.88E-02	2.04E-03	2.45	4.12E-02
SGPP2	cg14435109	223288714	223288837	2	TSS1500	5.72E-02	2.04E-03	−3.25	2.72E-03
SGPP2	cg07873848	223288635	223288758	2	TSS1500	6.25E-02	2.04E-03	−3.25	2.72E-03
SGPP2	cg11300809	223288637	223288760	2	TSS1500	7.03E-02	2.04E-03	−3.25	2.72E-03
SGPP2	cg10091265	223288331	223288454	2	TSS1500	7.16E-02	2.04E-03	−3.25	2.72E-03
SGPP2	cg16171484	223290515	223290638	2	Body	7.99E-02	2.04E-03	−3.25	2.72E-03
SMPD3	cg07735969	68418473	68418596	16	5'UTR	6.49E-02	2.04E-03	−3.52	2.63E-02
SRGN	cg18278184	70847430	70847553	10	TSS1500	8.69E-02	2.04E-03	3.49	4.83E-02
TNRC18	cg06947694	5389271	5389394	7	Body	−7.03E-02	2.04E-03	−2.58	1.12E-02
TNRC18	cg19679210	5354874	5354997	7	Body	6.58E-02	2.04E-03	−2.58	1.12E-02
TNXB	cg02657865	32077744	32077867	6	TSS1500	−8.95E-02	2.04E-03	2.82	2.92E-02
TNXB	cg13199127	32049196	32049319	6	Body	−8.53E-02	2.04E-03	2.82	2.92E-02
TNXB	cg25596754	32026610	32026733	6	Body	−7.77E-02	2.04E-03	2.82	2.92E-02
TNXB	cg00661399	32049177	32049300	6	Body	−7.60E-02	2.04E-03	2.82	2.92E-02
TNXB	cg24336152	32070785	32070908	6	5'UTR	−7.03E-02	2.04E-03	2.82	2.92E-02
TNXB	cg14669361	32038747	32038870	6	Body	−6.91E-02	0.00589	2.82	2.92E-02
TNXB	cg20858622	32015651	32015774	6	Body	−6.80E-02	9.84E-03	2.82	2.92E-02
TNXB	cg11493661	32016239	32016362	6	Body	−6.70E-02	5.63E-03	2.82	2.92E-02
TNXB	cg05956076	32074934	32075057	6	5'UTR	−6.55E-02	2.97E-02	2.82	2.92E-02
TNXB	cg16478197	32068181	32068304	6	5'UTR	−6.32E-02	2.04E-03	2.82	2.92E-02
TNXB	cg12493058	32052444	32052567	6	Body	5.59E-02	1.69E-02	2.82	2.92E-02
TNXB	cg20928974	32022642	32022765	6	Body	5.68E-02	1.97E-02	2.82	2.92E-02
TNXB	cg16662408	32053637	32053760	6	Body	5.95E-02	2.36E-02	2.82	2.92E-02
TNXB	cg23636802	32054441	32054564	6	Body	7.13E-02	2.36E-02	2.82	2.92E-02
TNXB	cg13606255	32053100	32053223	6	Body	7.94E-02	2.36E-02	2.82	2.92E-02
TNXB	cg13739666	32013974	32014097	6	TSS200	−7.87E-02	2.36E-02	2.82	2.92E-02
TNXB	cg26537323	32014059	32014182	6	TSS200	−7.60E-02	2.36E-02	2.82	2.92E-02
TNXB	cg18178844	32014100	32014223	6	TSS200	−6.24E-02	2.36E-02	2.82	2.92E-02
TNXB	cg25522795	32014096	32014219	6	TSS200	−5.09E-02	2.36E-02	2.82	2.92E-02
TNXB	cg15376677	32011687	32011810	6	Body	5.40E-02	2.36E-02	2.82	2.92E-02
TNXB	cg18340416	32010178	32010301	6	Body	5.89E-02	2.36E-02	2.82	2.92E-02
TNXB	cg20161227	31980836	31980959	6	5'UTR	−7.46E-02	2.36E-02	2.82	2.92E-02
TRIML1	cg04086012	189060900	189061023	4	1stExon	5.25E-02	2.36E-02	−3.35	2.19E-02
UNC13C	cg06530558	54304778	54304901	15	TSS1500	5.72E-02	2.36E-02	2.07	4.67E-02
VWA5B1	cg05805297	20617329	20617452	1	TSS200	7.04E-02	2.36E-02	4.25	1.29E-02
VWA5B1	cg04637372	20617456	20617579	1	5'UTR	7.54E-02	2.36E-02	4.25	1.29E-02
VWA5B1	cg25745746	20617452	20617575	1	5'UTR	8.04E-02	2.36E-02	4.25	1.29E-02
WNK4	cg06795963	40932359	40932482	17	TSS1500	−7.78E-02	2.36E-02	−3.68	1.54E-02
ZDHHC1	cg06139166	67440283	67440406	16	Body	−1.02E-01	2.36E-02	2.14	1.65E-02
ZDHHC1	cg08968184	67433636	67433759	16	Body	5.10E-02	2.36E-02	2.14	1.65E-02
ZFR2	cg09999510	3812479	3812602	19	Body	5.01E-02	2.36E-02	2.71	3.06E-02

*CHR, chromosome; DEG, differentially expressed gene; Δβ, difference of methylation between patients with obesity and healthy controls; DMP, differentially methylated position; START/END, position in Build 37; FC, log fold change*.

### Functional Annotation

**Figure 5:**
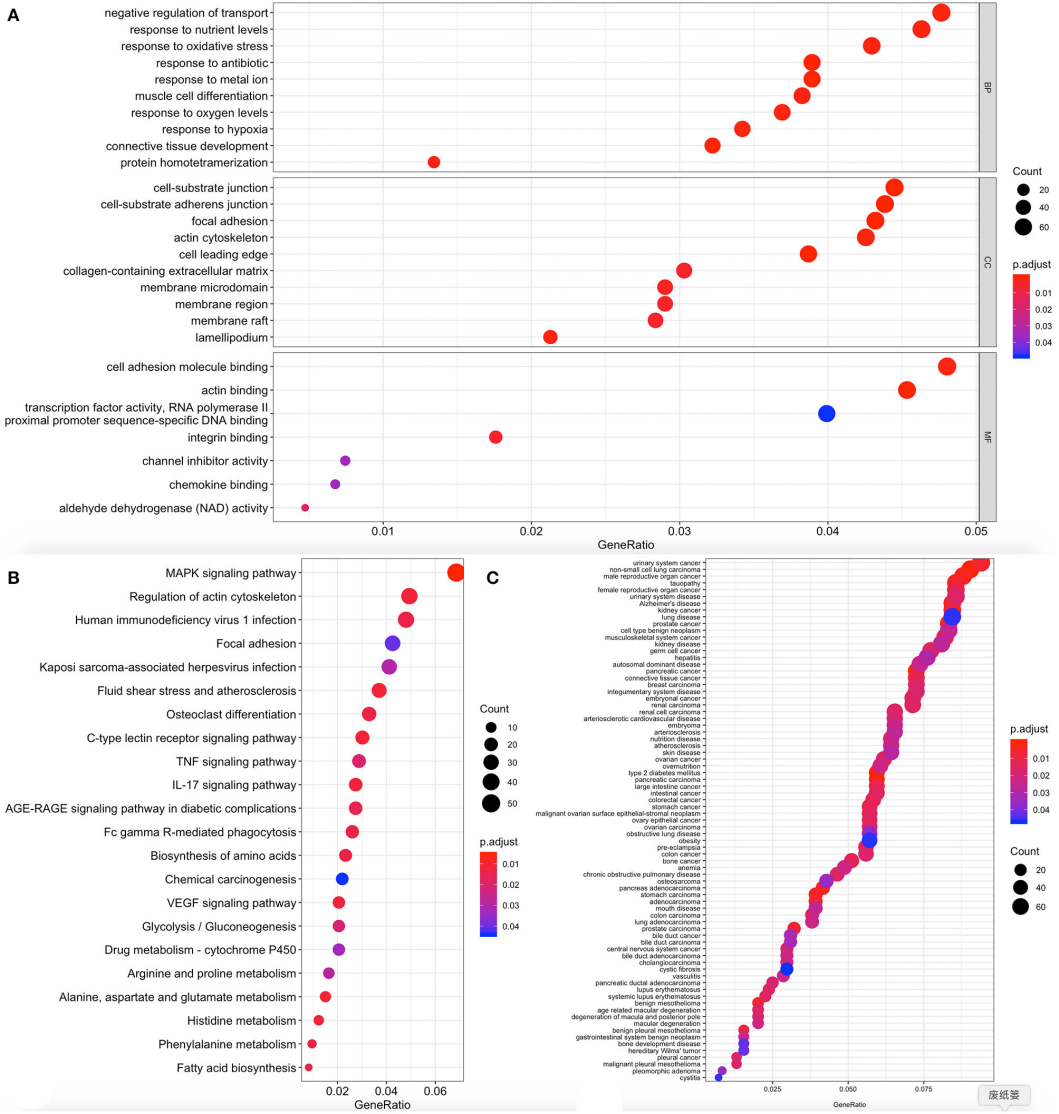
Functional enrichment of differentially expressed genes (DEGs). The x-axis shows the ratio number of genes and the y-axis shows the pathway terms. The -log10 (*P* value) of each term is colored according to the legend. **(A)** Gene Ontology; **(B)** Kyoto Encyclopedia of Genes and Genomes (KEGG) pathway analysis; **(C)** Disease Ontology.

The cluster profiler package in R for DO function, KEGG pathway enrichment, and GO analysis was used to clarify the role of the DEGs. Here, 104 cell components, seven molecular functions, and 329 biological processes were significantly enriched in GO (corrected *P* < 0.05; Figure 5A, Supplementary Table 1). Also, 22 pathways were enriched in KEGG, and 77 DO items were identified at *P* < 0.05 after correction (error detection rate, FDR <0.05). The results are shown in Figures 5B,C and Supplementary Tables 2, 3.

Enrichments including GO: 0061448 connective tissue development, GO: 0007584 nutritional response, GO: 0022600 digestive system process, GO: 0045444 adipocyte differentiation, hsa04010 MAPK signal pathway, hsa04657 IL-17 signal pathway, hsa0061 fatty acid biosynthesis, hsa04933 age-old signal pathway in diabetic complications, DOID: 9352 Type 2 diabetes mellitus; DOID: 374 nutritional diseases; DOID: 654 over nutrition; DOID: 9970 obesity have been previously reported to be related to obesity, and thus genes in these clusters were selected for further analysis.

### Construction of Protein–Protein Interaction Network and Identification of Key Genes

**Figure 6:**
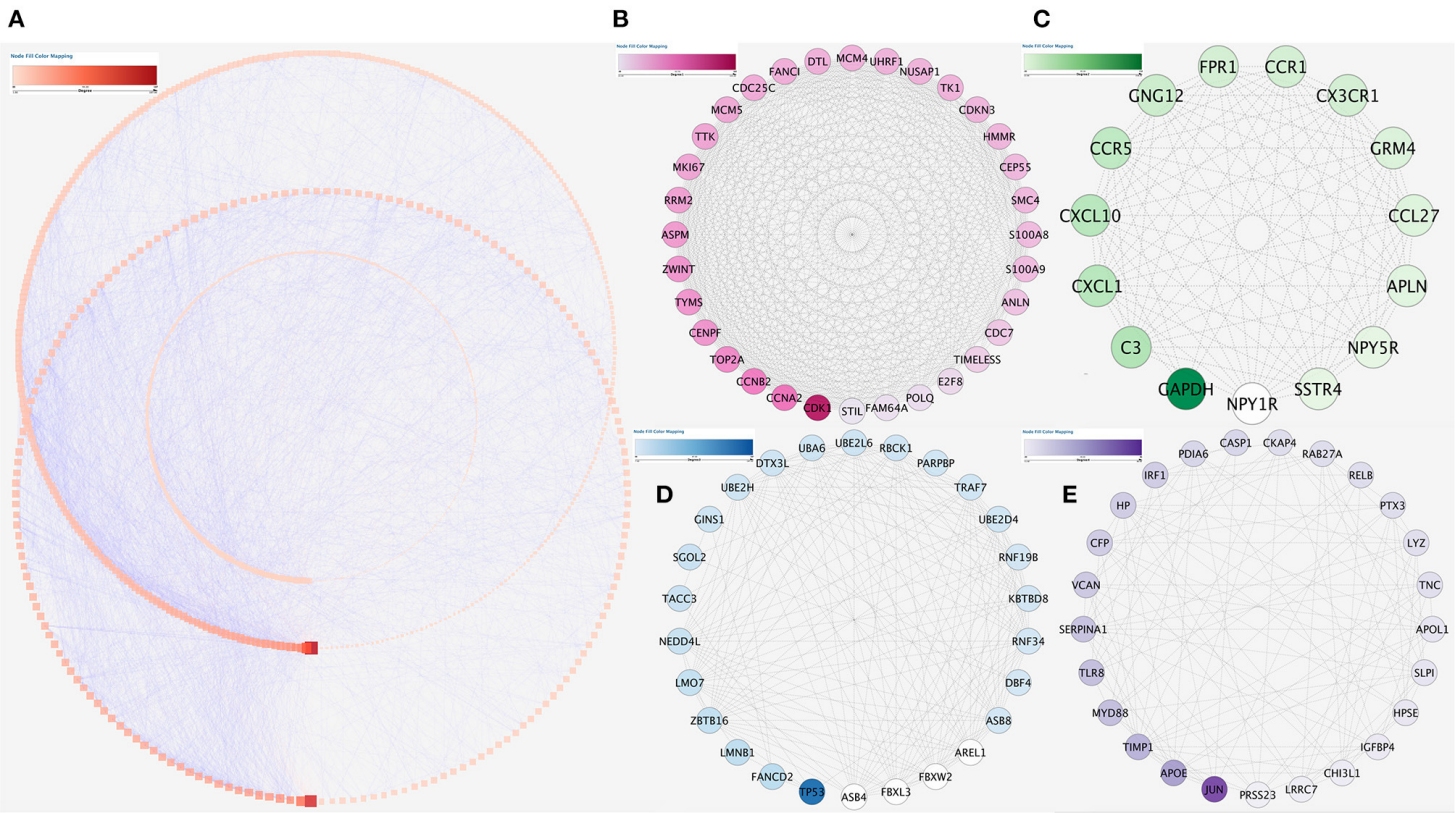
Protein–protein interaction network analysis. **(A)** Protein–protein interaction network of the module genes. Edge stands for the interaction between two genes. A degree was used for describing the importance of protein nodes in the network; darkness red shows a high degree and light presents a low degree. **(B–E)** The significant modules identified from the protein–protein interaction network using the molecular complex detection method with a score of >8.0. MCODE_B_ score = 22.166, MCODE_c_ score = 17.442, MCODE_D_ score = 9.278, and MCODE_E_ score = 8.963.

The string database was used to clarify gene interaction networks of the selected genes on Cytoscape, which revealed 5,598 pairs of proteins and 1,003 nodes when the cutoff value was set to a comprehensive score >0.9 (Figure 6A). MCODE analysis showed that the scores of the four modules were more than 10 (Figures 6B–E). Analysis of 98 genes in these four modules revealed that two of them were highly correlated and carried out submodule analysis to screen out GO, DO, and KEGG data. The two genes were S100 calcium-binding protein A8 (S100A8) and S100 calcium-binding protein A9 (S100A9).

### Hub Genes Validation

**Figure 7:**
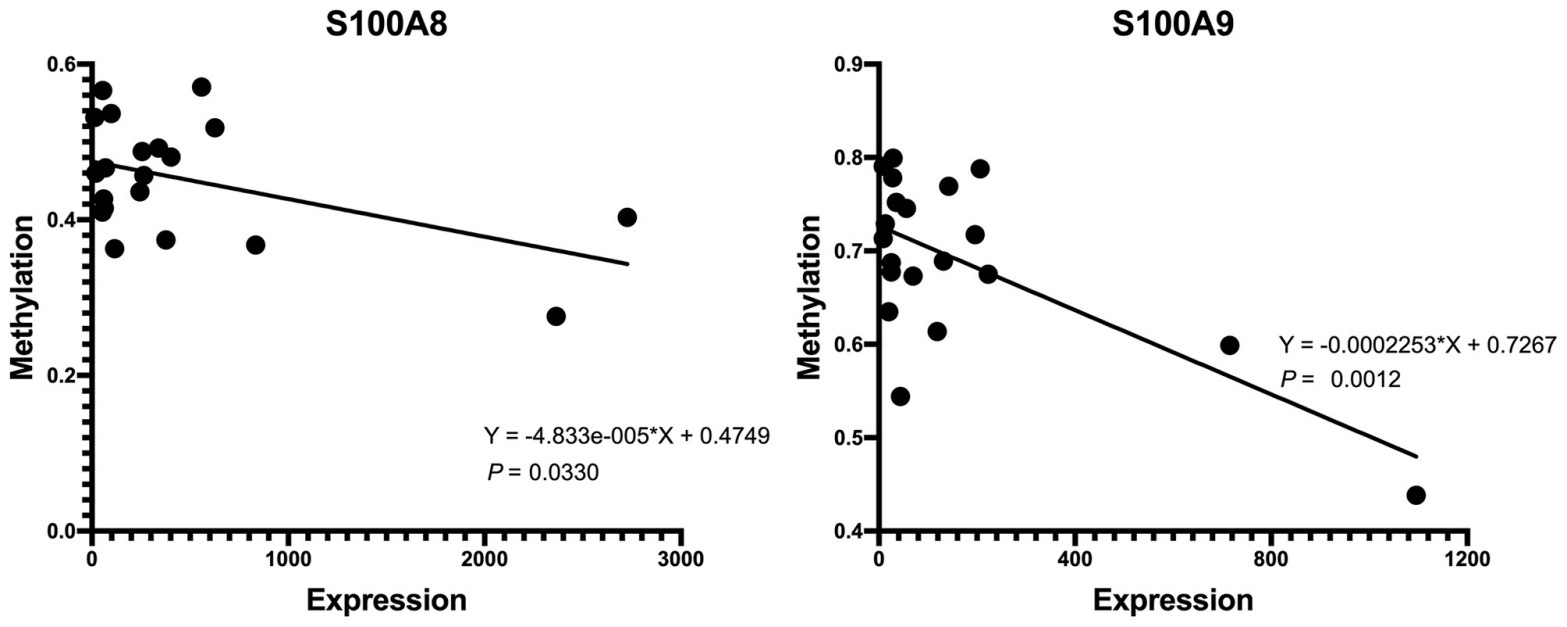
Correlation analysis of DNA methylation and mRNA expression.

**Figure 8:**
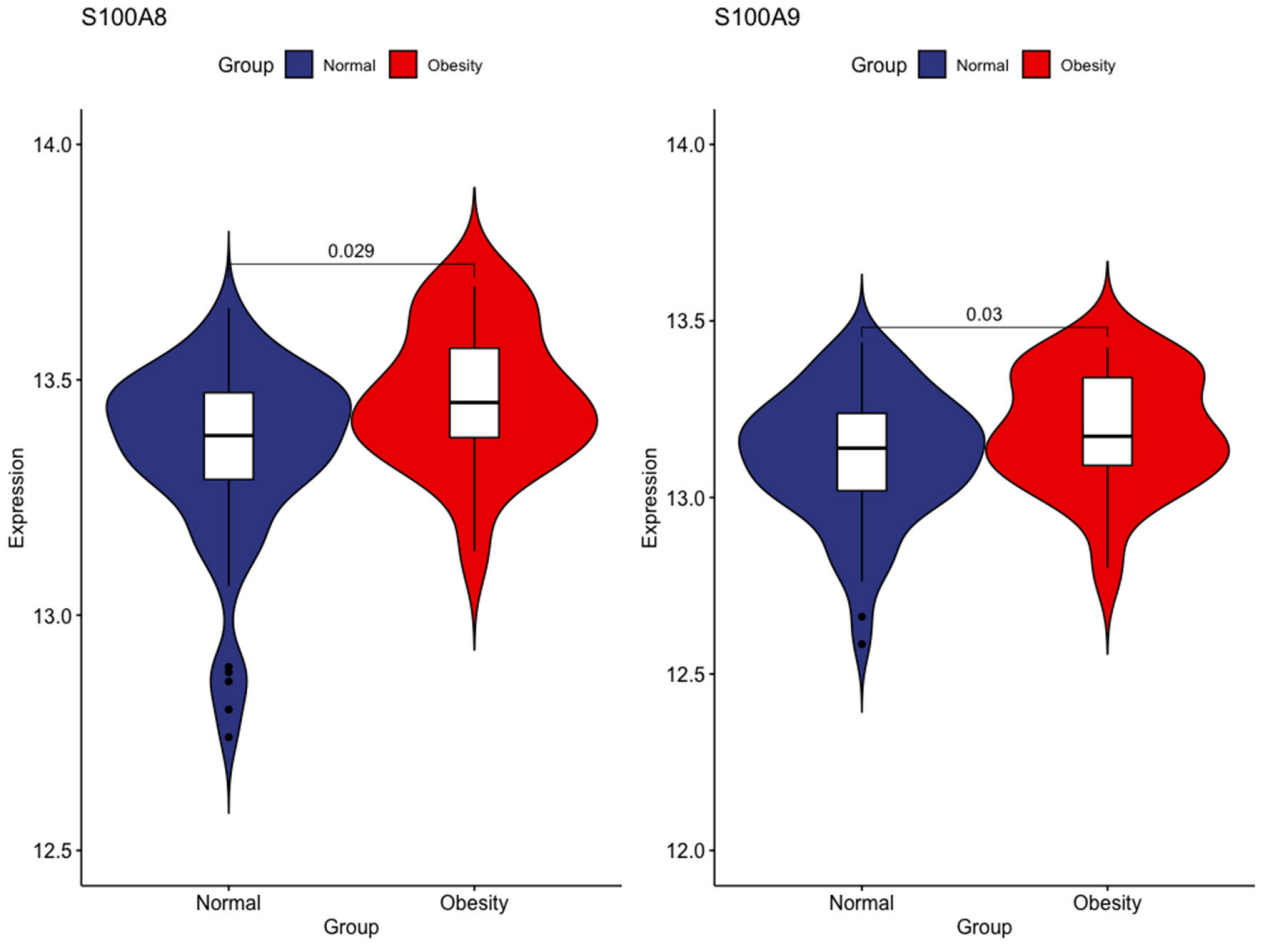
Verification of mRNA expression between obese and healthy samples in GSE109597.

First, we tested the relationship between S100A8 and S100A9 methylation and gene expression in the same 20 individuals in GSE88837 and GSE88940. We found that there was a negative correlation between methylation level and gene expression. The results also showed that the two correlation equation lines were statistically significant (*P* < 0.05; Figure 7). Validation in GSE109597 showed that the expression of S100A8 and S100A9 was significantly higher in obese vs. normal subjects (*P* < 0.05; Figure 8).

## Discussion

Emerging evidence has shown that obesity is not only a simple nutritional excess but also a metabolic disorder, which is the precursor of many metabolic diseases (18). More and more studies have confirmed that chronic inflammation is the result of the accumulation of fat cells caused by abnormal gene function (19). In the present study, the relationship between gene expression and obesity in 20 obese patients was analyzed using a gene-chip dataset. We found that the high expression of S100A8 and S100A9 was related to obesity. Also, a significant increase in S100A8 and S100A9 expression was observed in the validation microarray dataset of 84 samples. Whole-genome methylation analysis showed hypomethylation of the S100A8 and S100A9 promoters. Changes in methylation often lead to abnormal gene function and diseases and, in this case, may be an important cause of obesity.

Calprotectin is a heterodimer composed of S100A8 (calprotectin a, MRP8) and S100A9 (calprotectin B, MRP14) subunits, which are low-molecular weight members of S100 calprotectin subfamily (20). Previous studies have found that S100A8 and S100A9 are related to obesity, insulin resistance, and atherosclerosis (21). Sekimoto et al. (22) found that serum S100A8/A9 complex level was related to leukocyte count, BMI, subcutaneous fat area, and visceral fat area. At the same time, compared with lean mice, obese mice had higher S100A8 mRNA expression in the mature adipocyte component and obese mice had higher S100A9 expression in the matrix vascular component (22). Lylloff et al. (23) found that Roux-en-Y gastric bypass surgery (RYGB) significantly decreased BMI and circulating mRNA levels of S100A8 and S100A9 in obese patients. These findings are consistent with the present findings.

Epigenetics has become an intense topic of research in recent years. It refers to the regulation of gene expression without changing the basic structure of the gene. At present, epigenetics usually refers to histone modification and methylation of noncoding RNA and DNA (24, 25). Many studies have found that epigenetic mechanisms are associated with obesity. Sonne et al. (26) found changes in the methylation and expression of nine genes in epididymal adipocytes, including ehd2 and kctd15, which are known to be obesity-related genes, and a new candidate gene IRF8, which may be related to the 1/2 balance of immune type. Similarly, Bell (27) and van Dijk et al. (28) also explained the relationship between DNA methylation and obesity. It is worth noting that these two studies are comprehensive in content, more stringent in inclusion criteria, extensive in research content, and very reliable in conclusions (27, 28). Benton et al. (29) demonstrated the relationship between DNA methylation profiles of adipose tissue and weight loss before and after gastric bypass. This study provided a strong basis for future work and additional evidence for the role of DNA methylation in adipose tissue in obesity. The findings that the promoters of both the S100A8 and S100A9 genes were hypomethylated, in addition to their significantly increased expression in obese people, confirmed the validity of our findings. Other studies showed that methylation is closely associated with changes in adiposity. Brask et al. (26) found that obesity was associated with specific changes in adipocyte DNA methylation and gene expression. In general, obesity was associated with the global DNA hypomethylation significantly and epidermal fat exhibited more obesity-associated Differentially methylated regions (DMRs) than inguinal fat. Among genes that exhibited simultaneous changes in methylation and gene expression, some were involved in adipose tissue function in obese mice through hypomethylation-driven changes in expression. On the other hand, Riuzzi et al. (30) found that levels of S100A8/S100A9 were closely correlated with adipocyte size, and the mechanism may be related to the involvement in regulating the cell division cycle. Our study revealed that elevated levels of S100A8/S100A9 were closely associated with the development of obesity, while hypomethylation of the promoter region resulted in upregulation of S100A8/S100A9 expression.

There are a few limitations to this study. First of all, our data results only come from microarrays. Although it is clear that these two hub genes play roles in the pathogenesis of obesity, *in vitro* and *in vivo* studies are required to validate the relationship between these genes and obesity.

## Conclusion

In summary, two GEO microarrays were explored for differential methylation and gene expression as epigenetic factors of obesity. After the functional analysis, we selected two DEMGs from another microarray dataset (including 84 samples) for verification. Our results showed hypomethylation and upregulation of S100A8 and S100A9 expression in obese patients. Also, correlation analysis showed that DNA methylation can regulate gene expression and lead to obesity.

## Data Availability Statement

Publicly available datasets were analyzed in this study. This data can be found here: https://www.ncbi.nlm.nih.gov/geo/; Accession No.'s GSE88837, GSE88940 and GSE109597.

## Ethics Statement

All research protocols on this topic have been approved by the Ethics Committee of Guangxi Medical University (LSGXMU-2019-0028). The patients/participants provided their written informed consent to participate in this study.

## Author Contributions

NC conceived the study, participated in the design, undertook genotyping, performed the statistical analyses, and drafted the manuscript. LM participated in the design and analyzed the bioinformatics results. WL and DZ conceived the study and helped draft the manuscript. LH, JH, and WS collaborated for the genotyping. LL, YL, and HL carried out the epidemiological survey and collected the samples. SP and JP supervised the whole work. All authors read and approved the final manuscript.

## Conflict of Interest

The authors declare that the research was conducted in the absence of any commercial or financial relationships that could be construed as a potential conflict of interest.

## Supplementary Material

Supplementary Table 1 The analysis for Gene Ontology.

Supplementary Table 2 The analysis for KEGG pathway.

Supplementary Table 3 The analysis for Disease Ontology.

## References

[1] Jensen MD Ryan DH Apovian CM Ard JD Comuzzie AG Donato KA. 2013 AHA/ACC/TOS guideline for the management of overweight and obesity in adults: a report of the American College of Cardiology/American Heart Association Task Force on Practice Guidelines and The Obesity Society. Circulation. (2014) 129:S102–38. 10.1161/01.cir.0000437739.71477.ee 24222017 PMC5819889

[2] Collaborators GBDO Afshin A Forouzanfar MH Reitsma MB Sur P Estep K. Health effects of overweight and obesity in 195 countries over 25 years. N Engl J Med. (2017) 377:13–27. 10.1056/NEJMoa1614362 28604169 PMC5477817

[3] Global Burden of Metabolic Risk Factors for Chronic Diseases C Lu Y Hajifathalian K Ezzati M Woodward M Rimm EB. Metabolic mediators of the effects of body-mass index, overweight, and obesity on coronary heart disease and stroke: a pooled analysis of 97 prospective cohorts with 1.8 million participants. Lancet. (2014) 383:970–83. 10.1016/S0140-6736(13)61836-X 24269108 PMC3959199

[4] Lozano R Naghavi M Foreman K Lim S Shibuya K Aboyans V. Global and regional mortality from 235 causes of death for 20 age groups in 1990 and 2010: a systematic analysis for the Global Burden of Disease Study 2010. Lancet. (2012) 380:2095–128. 10.1016/S0140-6736(12)61728-0 23245604 PMC10790329

[5] Sayols-Baixeras S Subirana I Fernandez-Sanles A Senti M Lluis-Ganella C Marrugat J. DNA methylation and obesity traits: An epigenome-wide association study. The REGICOR study. Epigenetics. (2017) 12:909–16. 10.1080/15592294.2017.1363951 29099282 PMC5788444

[6] Smith ZD Meissner A. DNA methylation: roles in mammalian development. Nat Rev Genet. (2013) 14:204–20. 10.1038/nrg3354 23400093

[7] Xu X Su S Barnes VA De Miguel C Pollock J Ownby D. A genome-wide methylation study on obesity: differential variability and differential methylation. Epigenetics. (2013) 8:522–33. 10.4161/epi.24506 23644594 PMC3741222

[8] Rodriguez-Rodero S Menendez-Torre E Fernandez-Bayon G Morales-Sanchez P Sanz L Turienzo E. Altered intragenic DNA methylation of HOOK2 gene in adipose tissue from individuals with obesity and type 2 diabetes. PLoS ONE. (2017) 12:e0189153. 10.1371/journal.pone.0189153 29228058 PMC5724849

[9] Gautier L Cope L Bolstad BM Irizarry RA. affy–analysis of Affymetrix GeneChip data at the probe level. Bioinformatics. (2004) 20:307–15. 10.1093/bioinformatics/btg405 14960456

[10] Gentleman RC Carey VJ Bates DM Bolstad B Dettling M Dudoit S. Bioconductor: open software development for computational biology and bioinformatics. Genome Biol. (2004) 5:R80. 10.1186/gb-2004-5-10-r80 15461798 PMC545600

[11] Miao L Yin RX Pan SL Yang S Yang DZ Lin WX. Circulating miR-3659 may be a potential biomarker of dyslipidemia in patients with obesity. J Transl Med. (2019) 17:25. 10.1186/s12967-019-1776-8 30642348 PMC6332685

[12] Yu G Wang LG Han Y He QY. clusterProfiler. An R package for comparing biological themes among gene clusters. OMICS. (2012) 16:284–7. 10.1089/omi.2011.0118 22455463 PMC3339379

[13] Szklarczyk D Gable AL Lyon D Junge A Wyder S Huerta-Cepas J. STRING v11: protein-protein association networks with increased coverage, supporting functional discovery in genome-wide experimental datasets. Nucleic Acids Res. (2019) 47:D607–13. 10.1093/nar/gky1131 30476243 PMC6323986

[14] Miao L Yin RX Pan SL Yang S Yang DZ Lin WX. Weighted gene co-expression network analysis identifies specific modules and hub genes related to hyperlipidemia. Cell Physiol Biochem. (2018) 48:1151–63. 10.1159/000491982 30045016

[15] Shannon P Markiel A Ozier O Baliga NS Wang JT Ramage D. Cytoscape: a software environment for integrated models of biomolecular interaction networks. Genome Res. (2003) 13:2498–504. 10.1101/gr.1239303 14597658 PMC403769

[16] Bader GD Hogue CW. An automated method for finding molecular complexes in large protein interaction networks. BMC Bioinformatics. (2003) 4:2. 10.1186/1471-2105-4-2 12525261 PMC149346

[17] Miao L Yin RX Zhang QH Hu XJ Huang F Chen WX. Integrated DNA methylation and gene expression analysis in the pathogenesis of coronary artery disease. Aging. (2019) 11:1486–500. 10.18632/aging.101847 30844764 PMC6428103

[18] Force USPST Curry SJ Krist AH Owens DK Barry MJ Caughey AB. Behavioral weight loss interventions to prevent obesity-related morbidity and mortality in adults: US preventive services task force recommendation statement. JAMA. (2018) 320:1163–71. 10.1001/jama.2018.13022 30326502

[19] Yamaoka M Maeda N Takayama Y Sekimoto R Tsushima Y Matsuda K. Adipose hypothermia in obesity and its association with period homolog 1, insulin sensitivity, and inflammation in fat. PLoS ONE. (2014) 9:e112813. 10.1371/journal.pone.0112813 25397888 PMC4232416

[20] Catalan V Gomez-Ambrosi J Rodriguez A Ramirez B Rotellar F Valenti V. Increased levels of calprotectin in obesity are related to macrophage content: impact on inflammation and effect of weight loss. Mol Med. (2011) 17:1157–67. 10.2119/molmed.2011.00144 21738950 PMC3321803

[21] Yamaoka M Maeda N Nakamura S Mori T Inoue K Matsuda K. Gene expression levels of S100 protein family in blood cells are associated with insulin resistance and inflammation (Peripheral blood S100 mRNAs and metabolic syndrome). Biochem Biophys Res Commun. (2013) 433:450–5. 10.1016/j.bbrc.2013.02.096 23501102

[22] Sekimoto R Kishida K Nakatsuji H Nakagawa T Funahashi T Shimomura I. High circulating levels of S100A8/A9 complex (calprotectin) in male Japanese with abdominal adiposity and dysregulated expression of S100A8 and S100A9 in adipose tissues of obese mice. Biochem Biophys Res Commun. (2012) 419:782–9. 10.1016/j.bbrc.2012.02.102 22390934

[23] Lylloff L Bathum L Madsbad S Grundtvig JLG Nordgaard-Lassen I Fenger M. S100A8/A9 (Calprotectin), Interleukin-6, and C-Reactive protein in obesity and diabetes before and after Roux-en-Y gastric bypass surgery. Obes Facts. (2017) 10:386–95. 10.1159/000478097 28848164 PMC5644965

[24] Wahl S Drong A Lehne B Loh M Scott WR Kunze S. Epigenome-wide association study of body mass index, and the adverse outcomes of adiposity. Nature. (2017) 541:81–6. 10.1038/nature20784 28002404 PMC5570525

[25] Dick KJ Nelson CP Tsaprouni L Sandling JK Aissi D Wahl S. DNA methylation and body-mass index: a genome-wide analysis. Lancet. (2014) 383:1990–8. 10.1016/S0140-6736(13)62674-4 24630777

[26] Sonne SB Yadav R Yin G Dalgaard MD Myrmel LS Gupta R. Obesity is associated with depot-specific alterations in adipocyte DNA methylation and gene expression. Adipocyte. (2017) 6:124–33. 10.1080/21623945.2017.1320002 28481699 PMC5477735

[27] Bell CG. The epigenomic analysis of human obesity. Obesity. (2017) 25:1471–81. 10.1002/oby.21909 28845613

[28] van Dijk SJ Molloy PL Varinli H Morrison JL Muhlhausler BS Members of Epi S. Epigenetics and human obesity. Int J Obes. (2015) 39:85–97. 10.1038/ijo.2014.34 24566855

[29] Benton MC Johnstone A Eccles D Harmon B Hayes MT Lea RA. An analysis of DNA methylation in human adipose tissue reveals differential modification of obesity genes before and after gastric bypass and weight loss. Genome Biol. (2015) 16:8. 10.1186/s13059-014-0569-x 25651499 PMC4301800

[30] Riuzzi F Chiappalupi S Arcuri C Giambanco I Sorci G Donato R. S100 proteins in obesity: liaisons dangereuses. Cell Mol Life Sci. (2020) 77:129–47. 10.1007/s00018-019-03257-4 31363816 PMC11104817

